# Differences in glycated hemoglobin levels and cholesterol levels in individuals with diabetes according to *Helicobacter pylori* infection

**DOI:** 10.1038/s41598-021-87808-5

**Published:** 2021-04-19

**Authors:** Saeda Haj, Gabriel Chodick, Sophy Goren, Wasef Na’amnih, Varda Shalev, Khitam Muhsen

**Affiliations:** 1 Department of Epidemiology and Preventive Medicine, School of Public Health, Sackler Faculty of Medicine, Tel Aviv University, Ramat Aviv, 6139001 Tel Aviv, Israel; 2 Medical Division, Maccabi Health Services, Tel Aviv, Israel

**Keywords:** Endocrinology, Gastroenterology, Health care, Medical research, Risk factors

## Abstract

This study examined differences in glycated hemoglobin (HbA1c), fasting plasma glucose and cholesterol levels between *H. pylori* infected and uninfected persons with diabetes. Anonymized data of Maccabi Healthcare Services in Israel were analyzed, of 12,207 individuals (50.0% *H. pylori* positive) aged 25–95 years who underwent the urea breath test. The data included HbA1c, fasting plasma glucose and cholesterol levels. The inverse probability of treatment weighting approach was used to account for confounders. Differences between individuals who were *H. pylori* positive and negative, in HbA1c (> or ≤ 7.0%) and in cholesterol levels were assessed using weighted generalized estimating equations. For men, but not women, the likelihood of having HbA1c > 7.0% was increased in those infected than uninfected with *H. pylori*: prevalence ratio 1.11 (95% CI 1.00, 1.24), *P* = 0.04. For both sexes, total cholesterol (*P* = 0.004) and low-density lipoprotein (LDL) levels (*P* = 0.006) were higher among those infected than uninfected with *H. pylori*. No significant differences were found in glucose and HDL levels according to *H. pylori* infection. The results were consistent in unweighted multivariable analyses. In conclusion, *H. pylori* infection might be related to worse glycemic control in men, and higher total cholesterol and LDL cholesterol levels in both sexes.

## Introduction

*Helicobacter pylori* (*H. pylori*), a gram-negative bacterium, colonizes the stomach and causes chronic gastritis that mostly remains asymptomatic^1^. *H. pylori* causes ulcers in the stomach or duodenum only in a subset of infected people^2^, and is a risk factor for gastric adenocarcinoma and mucosa-associated lymphoid tissue lymphoma^1, 3, 4^.

*H. pylori* is involved in extragastric disorders such as iron deficiency anemia^5^ and idiopathic thrombocytopenic purpura^6^. We and others have shown positive associations of *H. pylori* infection and its-related gastroduodenal morbidity with diabetes mellitus^7–9^, metabolic syndrome^10, 11^ and glycated hemoglobin (HbA1c) levels^12^. Possible explanations for these relations include changes in gastric physiology induced by *H. pylori*. For example, *H. pylori* infection was shown to be related to ghrelin and leptin levels, two hormones that are expressed in the stomach and that play a major role in energy expenditure^13–15^. Chronic inflammation can contribute to the development of insulin resistance, which is pivotal in the pathophysiology of type 2 diabetes and metabolic syndrome^16^. Understanding the role of *H. pylori* infection in metabolic homeostasis and glycemic control in individuals with diabetes is paramount given the high burden of diabetes and its complications^17^ and the high *H. pylori* infection prevalence^1^.

Studies that examined differences in HbA1c and fasting glucose levels among individuals with diabetes according to *H. pylori* infection usually had small sample sizes^18–22^, and some included both individuals with type 1 and type 2 diabetes mellitus. Various methods of detecting *H. pylori* were used, including serological assays^20^, the urea breath test (UBT) and invasive examinations based on gastric biopsies^18, 19^. Evidence from these studies remains conflicting. One meta-analysis that included 14 studies (N = 1781 patients with diabetes) showed no significant difference in HbA1c levels between *H. pylori* infected and uninfected patients^23^, while another meta-analysis showed higher HbA1c levels among individuals who were *H. pylori* positive compared to negative; the weighted mean difference was 0.43, *P* = 0.02^24^.

The aim of this study was to compare HbA1c, fasting plasma glucose and cholesterol levels between *H. pylori* infected and uninfected persons with diabetes, independent of potential confounders. Our hypothesis was that compared to those uninfected, those infected with *H. pylori* would have higher fasting blood glucose, total cholesterol and low-density lipoprotein (LDL) levels; and lower high-density lipoprotein (HDL). Additionally, we hypothesized a greater proportion of individuals with higher HbA1c (> 7%) among those infected with *H. pylori.*

## Materials and methods

### Study design and population

A cross-sectional study was conducted using de-identified data from the computerized database of Maccabi Healthcare Services, the second largest health maintenance organization (HMO) in Israel. The study design was described elsewhere^7, 10^. Briefly, the study population comprised 147,936 persons who performed the UBT between 2002 and 2012. The age range at the UBT was 25–95 years; the mean was 42.8 years (standard deviation [SD] 12.7). Study exclusion criteria were determined based on factors that might affect the UBT result or metabolic control. Individuals were excluded if they purchased anti-*H. pylori* eradication therapy or proton pump inhibitors four weeks prior the UBT, had a cancer diagnosis within three years from the UBT or had documentation of bariatric surgery. Persons who purchased antibiotics for infections other than *H. pylori* were not excluded from the study. The current analysis was based on 12,207 (8.3%) patients with diabetes.

Diabetes was defined using the diabetes registry of Maccabi Healthcare Services. Persons with at least one of the following criteria were classified as having diabetes: 1) HbA1c ≥ 7.25%; 2) glucose ≥ 200 mg/dL; 3) diabetes diagnosis in the medical record (ICD-9 codes), and HbA1c ≥ 6.5% or glucose > 125 mg/dL; 4) two purchases of diabetes medications in the previous two months^25, 26^; these can be purchased by prescription only. The above data are routinely validated with primary care physicians.

*The dependent variables were* HbA1c (%), fasting plasma glucose (mg/dL), total cholesterol (mg/dL), LDL (mg/dL) and HDL (mg/dL) levels. Laboratory results of these tests were obtained from medical records. HbA1c was analyzed as a dichotomous variable; worse glycemic control was considered as HbA1c higher than 7%. Cholesterol and glucose levels were analyzed as continuous variables.

*The main independent variable was H. pylori infection:* positive or negative. *H. pylori* infection was defined based on the UBT result. The UBT was performed in fasting conditions. Individuals were asked to drink 75 mg of labeled urea with ^13^C, in 200 mL of orange juice. Breath samples were collected before ingestion of the labeled urea (baseline) and 30 min thereafter. Expired breath samples were analyzed by a mass spectrometer automated breath ^13^C analyzer. The ratio between ^12^C and ^13^C was measured at both time points in expired breath samples. The ratio of ^13^CO_2_ to ^12^CO_2_ in expired breath samples was determined and expressed as delta over baseline of ^13^CO_2_. *H. pylori* positivity was defined as ^13^CO_2_ delta over baseline > 3.5 per thousand^27^. The sensitivity and specificity of UBT were estimated at 96% and 93%, respectively^28^.

*Covariates* Potential confounders were selected based on evidence of their possible associations with *H. pylori* infection, glycemic control or cholesterol levels.

These included demographics: age (a continuous variable), sex, country of birth (classified as: Israel, the former Soviet Union, North Africa/Asia, Europe/Americas, and other/unknown) and residential socioeconomic status (SES)^29^. The presence of dyslipidemia was defined based on the International Classification of Disease codes-9th revision with clinical modifications (ICD-9-CM). Data were obtained on smoking (classified as ever, never, and unknown), body mass index (BMI) (weight in kilograms (kg)/height^2^ in meters (m)), and purchases of statins and diabetes medications (supplementary 1) one year prior to and one year after performing UBT. Each treatment was categorized as: 1) persistent use (three purchases with three-to-five-week interval between each two consecutive purchases); 2) non-persistent (purchases that did not meet the definition of persistent use), and 3) no purchases of these medications.

### Statistical analysis

Descriptive statistics using frequencies and percentages, or means and standard errors, were employed to describe the study sample. Initially we examined differences between individuals who tested positive and negative for *H. pylori,* in sociodemographic and clinical factors, using the Student’s *t* test for continuous variables, chi square tests for categorical variables, generalized linear models with binomial negative distribution and a log function link.

To account for potential confounders in the associations of *H. pylori* infection with glycemic control and cholesterol levels, we used the inverse probability of treatment weighting approach^30, 31^. We created a propensity score for the main independent variable^31–33^: *H. pylori* infection (positive or negative UBT) using the predicted probability of *H. pylori* positivity from a logistic regression model with the abovementioned covariates. Inverse probability weights were calculated using the propensity score created by weighting each participant in *H. pylori* positivity categories (*H. pylori* positive or *H. pylori* negative), inversely to his/her probability of being classified into these specific categories. This created a pseudopopulation, which achieved a balance between the independent variable in the distribution of a given covariate. We compared the proportions of individuals who were *H. pylori* positive and negative for each of the covariates examined, using weighted generalized estimating equations (GEE) with binomial negative distribution and a log function link. This provided a robust variance estimator^34^. Next, in an unweighted analysis, we assessed differences in the median levels of glucose and HbA1c between individuals who were *H. pylori* positive and *H. pylori* negative, using the Mann–Whitney *U* test. We used the chi square test to assess differences between the groups in the proportion with worse glycemic control (HbA1c > 7%). To account for confounders, as mentioned, the weighted GEE provides a robust variance estimator^34^, which was used to compare between individuals who were *H. pylori* positive and negative, the proportion with worse glycemic control (HbA1c > 7%). We also conducted unweighted multivariable generalized linear models that adjusted for confounders using the conventional approach. Prevalence ratios and 95% confidence intervals (CIs) were obtained from these models. We followed a similar strategy in comparing cholesterol levels between individuals who were *H. pylori* positive and *H. pylori* negative. Here, we initially using the Student's *t* test to assess differences between the groups in mean levels. To account for confounders, we used weighted GEE with linear models. We also conducted unweighted analysis by means of multiple linear regression models that included *H. pylori* infection, the independent variable, as a dummy variable; and sociodemographic and clinical variables (age, country of birth, the use of diabetes medications and statins, smoking and BMI) were covariates. Beta coefficients (the slope) and the corresponding 95% CI and R^2^ were obtained from the models. The assumptions of linear regression were met in the models of cholesterol levels. Differences between persons with and without *H. pylori* infection, in HbA1c, fasting glucose and cholesterol levels, were examined according to sociodemographic and clinical variables, using the Student's *t* test when comparing two groups, and one-way analysis of variance (ANOVA) when comparing three groups or more. The assumption of equal variance was examined using Levene's test; and when this was not met, we used the Welch's test. Correlations between age, and each of the dependent variables were examined using Pearson's correlation coefficient. Overall and sex-stratified analyses were performed. Interactions were examined of *H. pylori* infection with sex. Variables with missing data were handled using the missing indicator approach. Data were analysed using IBM-SPSS version 27 (IBM, Armonk, New York, USA). *P* < 0.05 was considered statistically significant. All statistical tests were 2-sided.

### Ethical consideration

The study protocol was approved by the Helsinki committee of Assuta Medical Center and the ethics committee of Tel Aviv University. Since this is a retrospective study that used coded (anonymized) administrative data from electronic medical records, the requirement for informed consent was waived by the Helsinki committee.

We confirm that all methods were performed in accordance with the relevant guidelines and regulations.

## Results

Of the 12,207 individuals with diabetes that comprised the cohort, 6,108 (50.0%) were positive for *H. pylori*. The mean age was significantly lower of those who were *H. pylori-*positive than *H. pylori*-negative: 54.4 years (SD = 11.7) vs. 57.9 (SD = 11.9). An inverse association was found between residential socioeconomic level and *H. pylori* infection. Individuals who were born in Europe and the Americas were less likely to have *H. pylori* infection than were those born in Israel. The proportion of individuals defined as persistent users of statins was lower among those who tested *H. pylori* positive than those who tested negative (50.4% vs. 59.9%). These differences were balanced in the weighted analysis (Table 1).

**Table 1:** Sociodemographic and clinical characteristics of individuals with diabetes, according to *H. pylori* positivity – unweighted and weighted analyses.

	Unweighted analysis				Weighted analysis			
	*H. pylori* positive N = 6108	*H. pylori* negative N = 6099	PR (95% CI)	*P* value^a^	*H. pylori* positive N = 6108	*H. pylori* negative N = 6099	PR (95% CI)	*P* value^b^
**Mean age, years (SD)**	54.4 (11.7)	57.9 (11.9)	0.99 (0.98, 0.99)	< 0.001	56.1 (11.8)	56.0 (12.1)	1.00 (0.99, 1.00)	0.88
**Sex**								
Male	2926 (47.9%)	2847 (46.7%)	1		47.2%	47.2%	1	
Female	3182 (52.1%)	3252 (53.3%)	0.97 (0.92, 1.04)	0.43	52.8%	52.8%	1.00 (0.96, 1.04)	0.99
**Residential SES**								
Low (1–5)	3111 (51.0%)	2583 (42.4%)	1		46.5%	46.4%	1	
Intermediate (6–7)	1541 (25.2%)	1685 (27.6%)	0.87 (0.81, 0.94)	0.0043	26.6%	26.6%	1.00 (0.96, 1.05)	0.99
High (8–10)	1167 (19.1%)	1552 (25.4%)	0.79 (0.72, 0.85)	6.0E-09	22.2%	22.3%	0.99 (0.95, 1.05)	0.95
Missing	289 (4.7%)	279 (4.6%)	0.93 (0.80, 1.08)	0.34	4.7%	4.7%	0.99 (0.92, 1.09)	0.98
**Country of birth**								
Israel	3196 (52.3%)	3080 (50.5%)	1		51.4%	51.5%	1	
Former Soviet Union	1838 (30.1%)	1659 (27.2%)	1.03 (0.96, 1.11)	0.38	28.4%	28.4%	1.00 (0.96 1.04)	0.96
North Africa/Asia	524 (8.6%)	480 (7.9%)	1.02 (0.91, 1.15)	0.67	8.3%	8.3%	1.00 (0.94, 1.07)	0.96
Europe/ Americas	347 (5.7%)	595 (9.8%)	0.72 (0.63, 0.82)	1.1E-06	7.8%	7.8%	1.00 (0.94, 1.08)	0.87
Other/unknown	203 (3.3%)	285 (4.7%)	0.82 (0.69, 0.97)	0.019	4.0%	4.0%	1.00 (0.91, 1.10)	0.97
**Mean BMI, kg/m**^**2**^**, SD**	30.4 (5.0)	30.1 (5.0)	1.01 (1.00, 1.01)	0.056	30.2 (5.0)	30.2 (5.1)	1.00 (0.99, 1.01)	0.99
**Smoking**				0.082				
Ever	1010 (16.5%)	859 (14.1%)	1		15.3%	15.3%	1	
Never	3591 (58.8%)	3742 (61.3%)	0.91 (0.83, 0.99)	0.025	60.0%	60.0%	0.99 (0.95, 1.05)	0.97
Unknown	1507 (24.7%)	1498 (24.6%)	0.93 (0.84, 1.02)	0.13	24.7%	24.7%	0.99 (0.94, 1.06)	0.95
**Dyslipidemia, yes**	4336 (71.0%)	4613 (75.6%)	0.89 (0.83, 0.95)	0.001	73.6%	73.5%	1.12 (1.08, 1.17)	0.93
**Statin**^c^								
Persistent use	3080 (50.4%)	3656 (59.9%)	0.82 (0.77, 0.89)	6.4E−08	55.2%	55.3%	1.00 (0.96, 1.04)	0.96
Not persistent use	1065 (17.4%)	864 (14.2%)	0.99 (0.91, 1.09)	0.93	15.8%	15.7%	1.00 (0.95, 1.06)	0.92
Never	1963 (32.1%)	1579 (25.9%)	1		29.0%	29.0%	1	
**Diabetes medications**^c^								
Persistent use	2041 (33.4%)	2357 (38.6%)	0.90 (0.84, 0.97)	0.003	36.0%	36.0%	1.02 (0.98, 1.06)	0.40
Not persistent use	1534 (25.1%)	1368 (22.4%)	1.02 (0.95, 1.11)	0.55	24.4%	22.9%	1.05 (1.01, 1.10)	0.043
Never	2533 (41.5%)	2,374 (38.9%)	1		39.6%	41.1%	1	

BMI: body mass index, CI: confidence interval, kg: kilogram, m: meters, PR: prevalence ratio; SD; standard deviation, SES: socioeconomic status.

^a^PR, 95% CI and *P* values were obtained using a univariable general linear model with negative binomial distribution; *P* value for the PR.

^b^PR, 95% CI and *P* values were obtained using weighted univariable generalized estimating equation models with negative binomial distribution; *P* value for the PR.

^c^Based on purchasing medications.

Differences in fasting blood glucose, HbA1c and cholesterol levels according to demographic and clinical factors appear in supplementary 2; Tables 1 and 2.

**Table 2:** Glucose and HbA1c levels of individuals with diabetes, according to *H. pylori* positivity, an-unweighted analysis.

	*H. pylori* positive	*H. pylori* negative	*P* value
**Overall**			
HbA1c (%), median, IQR	6.4 (1.1)	6.4 (1.1)	0.9^a^
HbA1c > 7.0%, N (%)	1071 (26.9%)	1025 (25.9%)	0.2^b^
Glucose level (mg/dL), median, IQR	118.0 (32.0)	119.0 (31.0)	0.2^a^
**Men**			
HbA1c (%), median, IQR	6.5 (1.3)	6.4 (1.1)	0.1^a^
HbA1c > 7.0%, N (%)	554 (30.1%)	484 (26.8%)	0.028^b,c^
Glucose level (mg/dL), median, IQR	120.0 (34.0)	120.0 (32.0)	0.4^a^
**Women**			
HbA1c (%), median, IQR	6.4 (1.0)	6.4 (1.1)	0.1^a^
HbA1c > 7.0%, N (%)	517 (24.2%)	541 (25.1%)	0.4^b,c^
Glucose level, median (mg/dL), IQR	117.0 (30.0)	119.0 (31.0)	0.2^a^

IQR: interquartile range.

^a^*P* value was obtained by the Mann–Whitney *U* test.

^b^Chi square test.

^c^Heterogeneity chi square = 4.074, *P* = 0.044 for the difference between men and women in the association between *H. pylori* infection and an HbA1c level > 7%.

### Associations of *H. pylori* infection with fasting blood glucose and HbA1c levels

Unweighted analysis showed no significant differences between individuals who were *H. pylori* positive and negative, in median HbA1c and glucose levels. A higher proportion of *H. pylori* infected men than uninfected men had worse glycemic control (HbA1c > 7%): 30.1% vs. 26.8%, *P* = 0.028. Such difference was not found in women (Table 2), heterogeneity chi square = 4.07, *P* = 0.044.

A weighted GEE model included worse control of diabetes (HbA1c > 7%) as the dependent variable. The independent variables were *H. pylori* infection, sex and the interaction term between sex and *H. pylori* infection. For men, but not women, this model showed a higher likelihood of worse glycemic control among individuals who tested positive versus negative for *H. pylori*: prevalence ratio 1.11 (95% CI 1.00, 1.24), *P* = 0.043. A similar but non-statistically significant (*P* = 0.083) result was obtained in an unweighted multivariable model that adjusted for sociodemographic and clinical factors (Table 3).

**Table 3:** The association of *H. pylori* infection with worse control of diabetes (HbA1c > 7%).

	Unweighted model 1^a^	*P* value	Unweighted model 2^a,b^	*P* value	Weighted model^c^	*P* value
	PR (95% CI)		PR (95% CI)		PR (95% CI)	
*H. pylori* positive vs. negative, men	1.12 (1.01, 1.24)	0.029	1.13 (0.98, 1.31)	0.093	1.11 (1.00, 1.24)	0.043
Interaction term: Sex (1 = women) by *H. pylori* positivity	0.86 (0.74, 0.99)	0.044	0.86 (0.70, 1.05)	0.13	0.88 (0.76, 1.02)	0.090
Women vs. men, *H. pylori* negatives	0.93 (0.84, 1.04)	0.2	0.90 (0.78, 1.04)	0.14	0.91 (0.82, 1.01)	0.082

CI: confidence intervals; PR: prevalence ratio.

^a^General linear model with negative binomial distribution and a log function link. The dependent variable was worse control of diabetes (HbA1c > 7%). The independent variables were *H. pylori* infection, sex and the interaction term sex (1 = women) by *H. pylori* infection.

^b^Model 2 is adjusted also for the variables age, body mass index (both continuous variables), residential socioeconomic rank, diagnosis of dyslipidemia, and the use of statins and diabetes medications.

^c^A generalized estimating equation model with negative binomial distribution and log function link. The dependent variable was worse control of diabetes (HbA1c > 7%), and the independent variables, *H. pylori* infection, sex and the interaction term sex (1 = women) by *H. pylori* infection.

No significant interaction was found between age and *H. pylori* infection, *P* = 0.17.

### Associations of *H. pylori* infection with cholesterol levels

Compared to persons uninfected, for persons who were infected with *H. pylori*, an unweighted analysis showed a higher mean total cholesterol level by 4.3 mg/dL (95% CI 2.5, 6.1), and a higher mean LDL level by 4.1 mg/dL (95% CI 2.6, 5.7). Similar findings were obtained in a stratified analysis by sex (Table 3).

In weighted GEE linear models, the differences between *H. pylori* infected and uninfected persons in total cholesterol and LDL cholesterol levels were attenuated and became non-statistically significant in men (Table 5). Adjustment for confounders using traditional linear regression models showed similar results to those obtained in the weighted models (Table 6). No significant difference was found between persons infected and uninfected with *H. pylori* in HDL level (Tables 4, 5, 6).

**Table 4:** Mean cholesterol levels of individuals with diabetes, according to *H. pylori* infection, an-unweighted analysis.

	*H. pylori* positive, Number	Mean (SE)	*H. pylori* negative, Number	Mean (SE)	Mean difference^a^ (95% CI)	*P* value^b^
**Overall**	6104		6103			
Total cholesterol (mg/dL)	4663	199.0 (0.65)	4653	194.7 (0.64)	4.3 (2.5, 6.1)	2.5E−06
LDL (mg/dL)	4605	120.0 (0.55)	4612	115.9 (0.55)	4.1 (2.6, 5.7)	1.3E−07
HDL (mg/dL)	4650	46.9 (0.17)	4637	47.4 (0.18)	0.5 (0.2, 1.0)	0.04
**Men**	2923		2850			
Total cholesterol (mg/dL)	2184	193.3 (0.98)	2129	186.9 (0.93)	6.4 (3.8, 9.0)	2.0E−06
LDL (mg/dL)	2144	117.7 (0.81)	2103	112.4 (0.81)	5.3 (3.0, 7.5)	5.0E−06
HDL (mg/dL)	2178	42.3 (0.21)	2119	42.6 (0.21)	− 0.3 (− 0.9, 0.3)	0.3
**Women**	3181		3253			
Total cholesterol (mg/dL)	2475	204.0 (0.86)	2528	201.3 (0.86)	2.7 (0.3, 5.1)	0.02
LDL (mg/dL)	2457	122.1 (0.75)	2513	118.8 (0.74)	3.3 (1.2, 5.3)	0.002
HDL (mg/dL)	2468	51.0 (0.24)	2522	51.5 (0.24)	− 0.5 (− 0.2, 1.2)	0.1

CI: confidence interval, HDL: high-density lipoproteins, LDL: low-density lipoproteins, SE: standard error.

^a^The mean difference between *H. pylori* infected and uninfected persons with diabetes.

^b^*P* value was obtained by Student's *t* test. The assumption of equal variance between *H. pylori* positive and negative individuals was met, as determined by Levene's F test, for all tested parameters.

**Table 5:** Cholesterol levels by sex -weighted generalized estimating equation models in individuals with diabetes, according to *H. pylori* infection^a^.

	*H. pylori* positive, Mean (SE)	*H. pylori* negative, Mean (SE)	Beta coefficient (95% CI)	*P* value
**Overall**^b^				
Total cholesterol (mg/dL)	198.2 (0.67)	195.5 (0.65)	2.7 (0.9, 4.5)	0.004
LDL (mg/dL)	119.1 (0.59)	116.9 (0.56)	2.2 (0.6, 3.8)	0.006
HDL (mg/dL)	47.3 (0.18)	47.1 (0.18)	0.2 (− 0.3, 0.7)	0.3
**Men**				
Total cholesterol (mg/dL)	191.5 (0.98)	188.9 (0.98)	2.6 (− 0.06, 5.4)	0.055
LDL (mg/dL)	116.2 (0.83)	114.2 (0.85)	2.0 (− 0.2, 4.3)	0.087
HDL (mg/dL)	42.5 (0.22)	42.3 (0.21)	0.2 (− 0.4, 0.8)	0.4
**Women**				
Total cholesterol (mg/dL)	203.9 (0.85)	201.2 (0.85)	2.8 (0.4, 5.2)	0.025
LDL (mg/dL)	121.6 (0.78)	119.2 (0.75)	2.4 (0.3, 4.5)	0.028
HDL (mg/dL)	51.4 (0.25)	51.1 (0.25)	0.3 (− 0.4, 0.9)	0.3

CI: confidence interval, HDL: high-density lipoproteins, LDL: low-density lipoproteins, SE: standard error.

^a^The weights were determined based on the inverse probability of treatment weighting approach. The weighted analysis was performed using generalized estimating equations with linear models. Beta coefficients, 95% CIs, *P* values, mean levels and standard errors in individuals with diabetes, according to *H. pylori* infection were obtained from these models.

^b^No significant interactions were found between sex and *H. pylori* infection (*P* = 0.9, for total cholesterol; and *P* = 0.8 for LDL and HDL models).

**Table 6:** Multivariable linear regression models of associations of *H. pylori* infection with cholesterol levels in individuals with diabetes, an unweighted analysis.

*H. pylori* infection (positive vs. negative)	Unstandardized beta coefficient (95% CI)	*P* value
**Overall**^a^		
Total cholesterol (mg/dL)^a1^	3.0 (1.3, 4.7)	0.001
LDL cholesterol (mg/dL)^a2^	2.5 (1.1, 3.9)	0.001
HDL cholesterol (mg/dL)^a3^	0.3 (− 0.1, 0.8)	0.1
**Men**^b^		
Total cholesterol (mg/dL)^b1^	3.5 (0.9, 6.0)	0.007
LDL cholesterol (mg/dL)^b1^	2.8 (0.6, 4.9)	0.011
HDL cholesterol (mg/dL)^b2^	0.3 (− 0.3, 0.9)	0.2
**Women**^b^		
Total cholesterol (mg/dL)^b3^	2.4 (0.2, 4.7)	0.035
LDL cholesterol (mg/dL)^b3^	2.1 (0.1, 4.1)	0.036
HDL cholesterol (mg/dL)^b2^	0.3 (− 0.4, 0.9)	0.3

^a^Each model was adjusted for age, body mass index (both continuous variables), sex, residential socioeconomic rank, diagnosis of dyslipidemia, and the use of statins and diabetes medications (dummy variables). ^a1^Adjusted R^2^ = 0.14; ^a2^Adjusted R^2^ = 0.13; ^a3^Adjusted R^2^ = 0.19.

^b^Each model was adjusted for age, body mass index (both continuous variables) residential socioeconomic rank, diagnosis of dyslipidemia, and the use of statins and diabetes medications (dummy variables); ^b1^Adjusted R^2^ = 0.14; ^b2^Adjusted R^2^ = 0.07; ^b3^Adjusted R^2^ = 0.12.

In all models, the values of variance inflation factor (VIF) ranged from 1.03 to 2.05, suggesting no multicollinearity.

Significant interactions were not found between age and *H. pylori* infection, and between sex and *H. pylori* infection.

## Discussion

We examined among persons with diabetes, differences in HbA1c, fasting plasma glucose and cholesterol levels, between those infected and not infected with *H. pylori*. To the best of our knowledge, this is the largest study to address this issue, with 12,207 individuals with diabetes.

The likelihood of having a high HbA1c (> 7.0%) was greater among *H. pylori* infected men than uninfected men. Such difference was not found in women (P for interaction 0.04). Mean total cholesterol and LDL cholesterol levels were higher among men infected with *H. pylori* than among individuals uninfected of both sexes. The results were consistent after adjustment for confounders using the inverse probability of treatment weighting approach and conventional multivariable models.

Previous studies that assessed differences in HbA1c and fasting glucose levels between *H. pylori* infected and uninfected persons with diabetes mostly reported no significant differences^18–21^. However, direct comparability between our and other studies might be limited due to methodological differences. Usually, men had a higher risk than women for *H. pylori*-related gastro-duodenal diseases such as peptic disease and gastric cancer^35, 36^. Therefore, the association of *H. pylori* with HbA1c levels in men only might not be surprising. Interestingly, Chen and Blaser^12^ reported positive associations between *H. pylori* sero-prevalence and HbA1c in a well-designed study of a large US general population sample; this supports our finding.

The observed difference in HbA1c levels by *H. pylori* infection in men is of modest magnitude. HbA1c is a measure of diabetes control and is associated with diabetic cardiovascular complications and mortality^37, 38^. Therefore, identifying modifiable factors that can affect HbA1c level is highly valuable. If *H. pylori* is causally related to HbA1c levels, then anti-*H. pylori* therapy is expected to affect HbA1c. Randomized controlled trials are needed to test this hypothesis. Even if the observed association between *H. pylori* and HbA1c in men with diabetes is not causal, our findings are still clinically important and can be useful in identifying persons at risk for less favorable glycemic control.

Interestingly, in both men and women, significantly higher total cholesterol and LDL cholesterol levels were found among *H. pylori* infected persons than uninfected ones. This result is in agreement with findings reported by Laurila et al.^39^. Vafaeimanesh et al.^21^ showed higher HDL cholesterol levels in *H. pylori* infected vs. uninfected persons with diabetes, but no significant differences in total cholesterol and LDL levels. A study from Germany showed no significant difference in total cholesterol, LDL and HDL levels among individuals with diabetes according to *H. pylori* sero-prevalence^40^. However, worse lipid profile was found in healthy persons infected with *H. pylori* than uninfected ones, when UBT was used to determine the presence of the infection^41^.

The association between cholesterol levels and cardiovascular disease is well established, and maintaining low LDL cholesterol is recognized as important for the prevention of cardiovascular disease^42^. Since diabetes is a risk factor for cardiovascular disease, the importance of maintaining optimal cholesterol levels is amplified in this context. Hence, our findings might serve as a basis for future clinical trials that assess whether *H. pylori* eradication can affect blood cholesterol levels in individuals with diabetes. Moreover, *H. pylori* infection might be a useful marker to identify persons with diabetes at risk for worse blood lipid levels.

The mechanism by which *H. pylori* infection might affect HbA1c and blood cholesterol levels is not fully clear, but might be related to the inflammation induced by the infection. *H. pylori* colonization in the stomach triggers a humoral and cellular immune response^43, 44^. The response of the cellular immune system is induced through T-helper-1 cells that release pro-inflammatory cytokines and activation of phagocytosis. *H. pylori* also activates T-helper 2 response and regulatory T-cells^43, 44^. Inflammatory agents such as cytokines and increased production of reactive oxygen species inhibit insulin signaling^16^.

Our study has some limitations. We used data from a large HMO database, which were collected for clinical care. The methods of collecting information on variables such as BMI and smoking may vary among physicians and nurses. Information was missing for some variables. For example, smoking status was unknown for 25% of the cohort. Data were collected of individuals who were referred to the UBT by their physicians. This implies that only those who had gastrointestinal symptoms were included.

Our study also has several strengths. The study was conducted using a large database of the second largest HMO in Israel, which insures about 25% of Israel's population; this is expected to increase the generalizability of the findings. The status of *H. pylori* infection was determined by UBT, which was shown to have high sensitivity and specificity, 96% and 93%, respectively^28^. In addition, levels of HbA1c, fasting blood glucose and cholesterol levels were tested in one laboratory. Lastly, the presence of diabetes was determined based on a combination of physician's diagnosis, laboratory results and purchasing of diabetes medications; this mitigates misclassification bias.

In conclusion, for men but not women, the likelihood of worse control of HbA1c was higher in persons who tested positive than negative for *H. pylori*. Higher total cholesterol and LDL cholesterol levels were found among men with *H. pylori* infection than among individuals of both sexes without infection. These associations were maintained after adjustment for potential confounders using a number of analytical methods. Our findings might have implications on glycemic control and the management of cholesterol levels in individuals with diabetes, and on the prevention of diabetes complications.

## Supplementary Information

Supplementary Information.

## Data Availability

The datasets generated during and/or analyzed during the current study are not publicly available due to local legal restriction and regulations.
