# Supplementary material for: Differences in glycated hemoglobin levels and cholesterol levels in individuals with diabetes according to Helicobacter pylori infection
Source: Sci Rep. 2021 Apr 19;11:8416. doi: 10.1038/s41598-021-87808-5 (PMC8055886; doi:10.1038/s41598-021-87808-5)
Supplement: Supplementary file 1 — Supplementary Information. [file 41598_2021_87808_MOESM1_ESM.pdf]

Differences in glycated hemoglobin levels and cholesterol levels in individuals with diabetes according to *Helicobacter pylori* infection

Saeda Haj[1], Gabriel Chodick[1,2], Sophy Goren[1], Wasef Na'amnih[1], Varda Shalev[1,2], Khitam Muhsen [1 *]

[1] Department of Epidemiology and Preventive Medicine, School of Public Health, Sackler Faculty of Medicine, Tel Aviv University, Tel Aviv, Israel

[2] Medical Division, Maccabi Health Services, Tel Aviv, Israel

Email addresses

Saeda Haj: saedahaj@gmail.com

Gabriel Chodick: hodik_g@mac.org.il

Sophy Goren: sophyg@post.tau.ac.il

Wasef Na'amnih: wasef25@yahoo.com

Varda Shalev: shalev_v@mac.org.il

Khitam Muhsen: kmuhsen@tauex.tau.ac.il

* Corresponding author

Khitam Muhsen, PhD

Department of Epidemiology and Preventive Medicine, School of Public Health, Sackler Faculty of Medicine, Tel Aviv University, Ramat Aviv, Tel Aviv, Israel 69978

Tel: 972-3-6405945; fax: 972-3-6409868

e-mail: kmuhsen@tauex.tau.ac.il

Supplementary 1

Diabetes medications

1. **Metformin:** Glucomin ,Glucophage ,Glufor.
2. **Sulfonylurea:**
    a. First generation: Tolbutamide (Orsinon)
    b. Second generation: Glibenclamide (Daonil ,Glibetic ,Gluben) , Glipizide (Gluco-Rite).
    c. Third generation: Glimepiride (amaryl).
3. **Meglitinies:** Repaglinide (Novonorm).
4. **Incretins:** Exenatide (Byetta), Liraglutide (Victosa).
5. **DPP-4 inhibitors:** Saxagliptin (Onglyza), Sitagliptin (Januvia, Januet), Vildagliptin (Eucreas, Galvus).
6. **α-glucosidase inhibitors:** Acarbose (Prandase, Acrose) pioglitazone (Actos).
7. **Thiazolidinedione:** Rosiglitazone (Rossini, Avandia).
8. **Insulin:**
    a. Rapid-Acting: Novorapid, Lispro, Humalog, Apidra.
    b. Short-Acting: Humulin R, Actrapid.
    c. Intermediate-Acting: Humulin NPH, Insulatard.
    d. Long-Acting: Lantus Levemir.
    e. Pre-Mixed: Humalogmix, Novomix, Mixtard.

Supplementary 2

Table 1: Differences in fasting blood glucose, HbA1c and cholesterol level according to demographic and clinical factors in men with diabetes [a]

	Fasting plasma glucose (mg/dL)	HbA1c (%)	Total cholesterol (mg/dL)	LDL (mg/dL)	HDL (mg/dL)
**Age** (Pearson's correlation coefficient [b])	0.02 (0.3)	-0.06 (0.01)	-0.20 (<0.001)	-0.20 (<0.001)	0.10 (<0.001)
**SES rank** [c]					
1-5	130.3 (42.4)	6.8 (1.4)	192.1 (46.2)	117.0 (38.0)	42.3 (10.0)
6-7	130.5 (39.8)	6.7 (1.4)	189.2 (44.1)	114.0 (37.8)	42.9 (9.5)
8-10	132.3 (43.7)	6.8 (1.3)	187.2 (40.8)	112.6 (35.7)	42.7 (9.9)
Missing	134.8 (47.3)	7.0 (1.6)	189.8 (43.4)	114.4 (37.1)	40.6 (8.4)
**Country of birth** [d]					
Israel	131.6 (44.9)	6.9 (1.4)	188.6 (43.3)	113.4 (36.8)	41.7 (9.8)
Former Soviet Union	130.1 (41.1)	6.7 (1.3)	196.8 (47.8)	120.3 (38.9)	43.7 (10.4)
North Africa/Asia	135.2 (42.7)	6.8 (1.5)	187.8 (42.8)	114.1 (37.0)	42.3 (9.7)
Europe/Americas	127.7 (33.5)	6.7 (1.1)	182.1 (40.2)	110.1 (36.2)	44.2 (10.0)
Other/unknown	129.2 (31.2	6.7 (1.1)	183.4 (36.3)	110.7 (32.2)	42.8 (9.0)
**BMI (kg/m^2)** [e]					
BMI <30	131.2 (42.0)	6.8 (1.4)	189.2 (44.1)	114.8 (37.7)	43.6 (10.2)
BMI ≥ 30	130.7 (42.7)	6.8 (1.3)	191.6 (45.0)	115.6 (37.3)	40.8 (8.9)
Missing	135.0 (48.1)	6.6 (1.0)	181.6 (26.5)	108.3 (26.5)	43.0 (9.3)
**Smoking** [f]					
Ever	132.8 (48)	6.9 (1.5)	194.6 (46.5)	118.6 (38.8)	41.4 (9.1)
Never	130.7 (40.1)	6.8 (1.3)	187.7 (43.7)	113.2 (36.7)	43.0 (10.0)
Missing	130.0 (42.0)	6.8 (1.4)	192.0 (43.5)	116.6 (37.6)	42.3 (10.0)
**Dyslipidemia** [g]					
No	131.6 (44.3)	6.9 (1.5)	178.9 (37.7)	107.5 (31.3)	43.7 (10.8)
Yes	130.8 (41.6)	6.8 (1.3)	194.0 (45.8)	117.7 (39.0)	42.0 (9.3)
**Statins use** [h]					
Persistent	132.4 (40.0)	6.8 (1.2)	185.1 (46.5)	109.8 (39.0)	42.9 (9.7)
Non-persistent	136.6 (49.4)	7.1 (1.6)	206.0 (44.0)	129.9 (37.9)	41.6 (9.6)
No purchases	125.4 (41.5)	6.6 (1.5)	190.8 (37.7)	117.0 (31.1)	42.0 (10.0)
**Diabetes medications use** [i]					
Persistent	143.5 (49.1)	7.1 (1.4)	180.6 (44.7)	105.1 (35.8)	42.4 (10.1)
Non-persistent	144.5 (49.3)	7.2 (1.6)	184.0 (44.3)	110.2 (38.7)	41.6 (9.3)
No purchases	113.2 (19.2)	6.1 (0.7)	201.5 (41.5)	126.2 (35.0)	43.1 (9.7)

BMI: Body mass index: HDL: high-density lipoproteins, kg: kilogram, LDL: low-density lipoproteins, m: meters, SES rank: Socioeconomic status rank of city/town of residence: 1-5 represent low SES, 6-7 intermediate SES and 8-10 high SES.

[a] Average and standard deviation in parentheses, unless stated otherwise. [b] Pearson correlation coefficient and statistical significance between parentheses. [c] P value by Welch's analysis of variance for the difference between the groups in levels of glucose (P=0.3), HbA1c (P=0.13) and by parametric ANOVA for total cholesterol (P=0.031), LDL cholesterol (P=0.018) and HDL cholesterol (P=0.015).

[d] P value by Welch's analysis of variance for the difference between the groups in levels of glucose (P=0.07), HbA1c (P=0.04) total cholesterol (P<0.001), LDL cholesterol (P<0.001) and HDL cholesterol (P<0.001).

[e] P value by parametric ANOVA for the difference between the groups in levels of glucose (P=0.8), HbA1c (P=0.7) and Welch's analysis of variance for differences in total cholesterol (P=0.038), LDL cholesterol (P=0.2) and HDL cholesterol (P<0.001).

[d] P value by Welch's analysis of variance for the difference between the groups in levels of glucose (P=0.3), HbA1c (P=0.13) and parametric ANOVA for the difference in total cholesterol (P<0.001) and LDL cholesterol (P=0.001) and HDL cholesterol (P<0.001).

[g] P value the Student's *t* test for the difference between the groups in levels of glucose (P=0.5), and Welch's t test for the difference in HbA1c (P=0.2) total cholesterol (P<0.001), LDL cholesterol (P<0.001) and HDL cholesterol (P<0.001).

[h] P value by Welch's analysis of variance for the difference between the groups in levels of glucose (P<0.001), HbA1c (P<0.001) total cholesterol (P<0.001), LDL cholesterol (P<0.001) and HDL cholesterol (P=0.002).

[i] P value by Welch's analysis of variance for the difference between the groups in levels of glucose (P<0.001), HbA1c (P<0.001) and parametric ANOVA for the difference in total cholesterol (P<0.001), LDL cholesterol (P<0.001) and HDL cholesterol (P<0.001).

Table 2: Differences in fasting blood glucose, HbA1c and cholesterol level according to demographic and clinical factors in women with diabetes [a]

	Fasting plasma glucose (mg/dL)	HbA1c (%)	Total cholesterol (mg/dL)	LDL (mg/dL)	HDL (mg/dL)
**Age** (Pearson's correlation coefficient [b])	0.03 (0.1)	-0.05 (0.03)	-0.05 (0.01)	-0.10 (0.001)	0.10 (0.001)
**SES rank** [c]					
1-5	126.9 (35.7)	6.7 (1.2)	203.7 (43.1)	121.5 (37.7)	50.9 (12.1)
6-7	127.6 (36.6)	6.7 (1.2)	201.8 (41.5)	120.0 (36.8)	51.3 (12.2)
8-10	128.1 (36.6)	6.7 (1.1)	201.7 (44.1)	119.1 (37.0)	52.2 (12.1)
Missing	131.5 (43.2)	7.0 (1.5)	200.1 (44.8)	118.5 (38.3)	50.4 (12.3)
**Country of birth**					
Israel	127.7 (37.7)	6.7 (1.3)	199.2 (41.9)	118.4 (36.1)	50.1 (11.7)
Former Soviet Union	127.0 (34.3)	6.7 (1.1)	206.3 (44.0)	123.5 (39.0)	52.3 (12.3)
North Africa/Asia	127.3 (37.3)	6.7 (1.2)	199.0 (41.6)	116.6 (34.7)	51.2 (12.1)
Europe/Americas	129.5 (38.7)	6.7 (1.2)	206.8 (42.2)	121.5 (35.9)	53.5 (13.1)
Other/unknown	126.6 (38.1)	6.7 (1.3)	200.8 (44.9)	117.3 (38.1)	52.8 (12.8)
**BMI ($kg/m^{2)}$** [e]					
BMI <30	125.1 (35.8)	6.7 (1.2)	203.7 (43. 1)	121.8 (37.4)	53.0 (12.5)
BMI ≥ 30	129.2 (36.5)	6.7 (1.2)	201.5 (42.1)	119.1 (37.1)	49.8 (11.5)
Missing	145.1 (56.3)	6.9 (1.3)	216.5 (74.3)	127.2 (46.8)	51.7 (16.7)
**Smoking** [f]					
Ever	125.3 (37.8)	6.7 (1.1)	203.7 (43.8)	121.9 (36.8)	49.4 (12.3)
Never	128.2 (36.6)	6.7 (1.2)	203 (42.8)	120.5 (37.7)	51.7 (12.1)
Missing	126.6 (35.8)	6.7 (1.2)	201.3 (43.2)	119.7 (36.7)	50.8 (12.0)
**Dyslipidemia** [g]					
No	128.1 (36.1)	6.7 (1.2)	208.4 (44.0)	124.6 (38.7)	51.3 (12.0)
Yes	125.8 (37.5)	6.6 (1.2)	185.9 (35.0)	108.2 (30.0)	51.2 (12.4)
**Statins use** [h]					
Persistent	130.4 (36.3)	6.8 (1.1)	201.1 (44.6)	117.8 (39.2)	51.8 (12.1)
Non-persistent	129.0 (38.1)	6.8 (1.4)	220.2 (43.2)	136.7 (37.0)	50.3 (11.4)
No purchases	120.5 (34.9)	6.4 (1.2)	195.5 (36.2)	116.7 (30.4)	50.5 (12.5)
**Diabetes medications use**					
Persistent	138.4 (39.7)	7.0 (1.2)	194.5 (41.5)	112.2 (36.9)	50.9 (12.0)
Non-persistent	137.4 (44.6)	7.0 (1.5)	201.8 (44.9)	119.4 (38.1)	50.5 (12.5)
No purchases	110.7 (15.3)	6.1 (0.6)	211.2 (41.6)	129.4 (35.2)	52.0 (12.1)

BMI: Body mass index: HDL: high-density lipoproteins, kg: kilogram, LDL: low-density lipoproteins, m: meters, SES rank: Socioeconomic status rank of city/town of residence: 1-5 represent low SES, 6-7 intermediate SES and 8-10 high SES. [a] Average and standard deviation in parentheses, unless stated otherwise. [b] Pearson correlation coefficient and statistical significance between parentheses. [c] P value by Welch's analysis of variance for the difference between the groups in levels of glucose (P=0.4), HbA1c (P=0.09) and by parametric ANOVA for total cholesterol (P=0.3), LDL cholesterol (P=0.2) and HDL cholesterol (P=0.024). [d] P value by Welch's analysis of variance for the difference between the groups in levels of glucose (P=0.8), HbA1c (P=0.5) total cholesterol (P<0.001), LDL cholesterol (P<0.001) and HDL cholesterol (P<0.001). [e] P value by Welch's analysis of variance for the difference between the groups in levels of glucose (P<0.001), HbA1c (P=0.2) total cholesterol (P=0.019), LDL cholesterol (P=0.019) and HDL cholesterol (P<0.001).

[f] P value by ANOVA for the difference between the groups in levels of glucose (P=0.15), HbA1c (P=0.4) total cholesterol (P=0.3), LDL cholesterol (P=0.5) and HDL cholesterol (P<0.001).

[g] P value the Student's *t* test for the difference between the groups in levels of glucose (P=0.057) and HbA1c (P=0.034) and Welch's t test for the difference in total cholesterol (P<0.001), LDL cholesterol (P<0.001) and HDL cholesterol (P=0.9).

[h] P value by Welch's analysis of variance for the difference between the groups in levels of glucose (P<0.001), HbA1c (P<0.001) total cholesterol (P<0.001), LDL cholesterol (P<0.001) and HDL cholesterol (P<0.001).

i P value by Welch's analysis of variance for the difference between the groups in levels of glucose (P<0.001), HbA1c (P<0.001) and parametric ANOVA for the difference in total cholesterol (P<0.001), LDL cholesterol (P<0.001) and HDL cholesterol (P=0.13).
